# Maximum emergency department overcrowding is correlated with occurrence of unexpected cardiac arrest

**DOI:** 10.1186/s13054-020-03019-w

**Published:** 2020-06-06

**Authors:** June-sung Kim, Hyun-Jin Bae, Chang Hwan Sohn, Sung-Eun Cho, Jeongeun Hwang, Won Young Kim, Namkug Kim, Dong-Woo Seo

**Affiliations:** 1 Department of Emergency Medicine, University of Ulsan, College of Medicine, Asan Medical Center, Seoul, Republic of Korea; 2 Promedius Inc., Seoul, Republic of Korea; 3 Nursing Department, Asan Medical Center, Seoul, Republic of Korea; 4 Department of Convergence Medicine, University of Ulsan, College of Medicine, Asan Medical Center, 88, Olympic-ro 43-gil, Songpa-gu, Seoul, 05505 Republic of Korea; 5 Department of Emergency Medicine, Biomedical Informatics, University of Ulsan, College of Medicine, Asan Medical Center, 88, Olympic-ro 43-gil, Songpa-gu, Seoul, 05505 Republic of Korea

**Keywords:** Emergency department crowding, In-hospital cardiac arrest, Out-of-hospital cardiac arrest, Quality control

## Abstract

**Background:**

Emergency department overcrowding negatively impacts critically ill patients and could lead to the occurrence of cardiac arrest. However, the association between emergency department crowding and the occurrence of in-hospital cardiac arrest has not been thoroughly investigated. This study aimed to evaluate the correlation between emergency department occupancy rates and the incidence of in-hospital cardiac arrest.

**Methods:**

A single-center, observational, registry-based cohort study was performed including all consecutive adult, non-traumatic in-hospital cardiac arrest patients between January 2014 and June 2017. We used emergency department occupancy rates as a crowding index at the time of presentation of cardiac arrest and at the time of maximum crowding, and the average crowding rate for the duration of emergency department stay for each patient. To calculate incidence rate, we divided the number of arrest cases for each emergency department occupancy period by accumulated time. The primary outcome is the association between the incidence of in-hospital cardiac arrest and emergency department occupancy rates.

**Results:**

During the study period, 629 adult, non-traumatic cardiac arrest patients were enrolled in our registry. Among these, 187 patients experienced in-hospital cardiac arrest. Overall survival discharge rate was 24.6%, and 20.3% of patients showed favorable neurologic outcomes at discharge. Emergency department occupancy rates were positively correlated with in-hospital cardiac arrest occurrence. Moreover, maximum emergency department occupancy in the critical zone had the strongest positive correlation with in-hospital cardiac arrest occurrence (Spearman rank correlation *ρ* = 1.0, *P* < .01). Meanwhile, occupancy rates were not associated with the ED mortality.

**Conclusion:**

Maximum emergency department occupancy was strongly associated with in-hospital cardiac arrest occurrence. Adequate monitoring and managing the maximum occupancy rate would be important to reduce unexpected cardiac arrest.

## Introduction

Emergency department (ED) overcrowding is a global phenomenon with complex causes that are not fully understood [1, 2]. The imbalance of supply and demand in ED capacity can result in hazardous effects both on physicians and patients [3], and previous studies have found that overcrowded ED conditions increased physician decision-making time [4], led to more medication errors [5], decreased quality of care [6], and increased mortality during hospital stay [7, 8]. These potential dangers can be particularly detrimental for patients who needed time-critical interventions to effectively treat emergency conditions such as acute stroke [9], acute respiratory failure [10], and septic shock [11].

In-hospital cardiac arrest is a critical situation requiring prompt intervention and continues to be a major public health burden [12, 13]. A previous study found that the quality of cardiopulmonary resuscitation (CPR) of out-of-hospital cardiac arrest (OHCA) was not associated with overcrowding because emergency medical services could alert the ED CPR team of incoming OHCA patients, allowing time for the team to activate [14]. Compared to OHCA, many in-hospital cardiac arrests (IHCAs), especially during ED stay, are unexpected but considered to be preventable if early identification of at-risk patients and adequate interventions are possible [15]. However, there is limited evidence as to whether ED crowding influences the occurrence or outcomes of IHCA. We hypothesized that ED overcrowding correlates with IHCA occurrence. To test this hypothesis, we used a prospective registry of in-hospital cardiac arrest to evaluate the association between the ED occupancy rate in various timing and unexpected cardiac arrest.

## Method

### Study design

This was a single-center, observational, registry-based cohort study conducted at the ED of a university-affiliated teaching hospital in Seoul, Korea, which receives approximately 110,000 patients per year. We retrospectively analyzed registry data of all consecutive adult (age ≥ 18 years), non-traumatic IHCA patients which occurred only during the ED stay but not after the admission. The study period was between January 2014 and June 2017. Patients were excluded if they were younger than 18 years, had do-not-resuscitate status, were dead upon arrival at the ED, had arrest after admission, or had re-arrest after the return of spontaneous circulation (ROSC) occurred before ED arrival. Our institutional review board approved the study and waived the requirement for informed consent (study number S2017-1004).

Our ED had triage and 7 zones with a total of 65 beds, including 2 beds in the CPR zone, 3 beds in the isolation rooms for airborne-transmitted disease, 37 beds in the intensive care zone, and 23 in the zone for boarding or ambulatory care. One staff usually examined patients just after arrival (i.e., within around 5 min) in triage and classified them based on severity. South Korea currently has used the Korean Triage and Acuity Scale (KTAS) since 2016 [16]. For simplification of analysis, we classified the ED zone into two zones: the critical zone for patients in need of CPR, isolation, or intensive care and the urgent zone for patients intended to be admitted to the hospital, to be discharged to home or another facility, or to receive ambulatory care. During the study period, the ED had an average of 16 board-certified emergency physicians, 24 residents, and 90 nurses. During each shift, two board-certified emergency medicine physicians on duty worked in each area (i.e., one for the critical care and one for the urgent area) at the same time with 2 to 3 residents and 2 to 4 interns. The study facility had 24-h consultants including cardiologists, vascular surgeons, neurologists, neurosurgeons, interventional radiologists, and orthopedic surgeons.

### Data collection

All patients presenting with IHCA were registered in the CPR registry of our hospital. For each patient, an emergency physician on duty recorded a CPR report using the Utstein style, and the data were verified and entered into the web-based registry by the principal investigator.

Data included demographic characteristics, medical history, and characteristics of CPR, such as presumed causes of arrest, do-not-resuscitate (DNR) order during or after CPR, initial reported rhythm, time of the first defibrillation, time of the first epinephrine injection, time of endotracheal intubation, total CPR duration, and whether ROSC occurred. The registry also contained information about hospital treatment, such as hypothermia, percutaneous coronary intervention, and extracorporeal membrane oxygenation, which were determined to be performed by physicians on duty. Furthermore, clinical outcomes, including death in ED, hospital length of stay, and neurologic status at discharge, were extracted. Hospital length of stay included the duration of the length of stay from ED arrival to hospital discharge. Achievement of sustained ROSC was declared when patients had a noticeable pulse for longer than 20 min. Neurologic status was quantified based on cerebral performance category (CPC) scales at the time of hospital discharge, and CPC 1–2 were considered as favorable neurologic outcomes [17].

We used ED occupancy rate as an overcrowding index. ED occupancy rate was defined as the ratio of the total number of ED patients to the number of beds in the ED [18]. Although there is no universal consensus on overcrowding measurement, ED occupancy rate is one of the most promising quantifiable methods [19]. It is essential that the ED occupancy rate is automatically updated periodically since this data has a time-series nature; our electronic medical record system automatically collects variables necessary for ED occupancy rate calculation at 1-h intervals. Previous studies using ED occupancy rate only measured occupancy at ED presentation [20–22]. To evaluate if other times during an ED cardiac arrest were associated with crowding levels, we measured ED occupancy rates of IHCA cases at presentation, at time of arrest, at time of maximum occupancy, and at the average during ED stay. We then calculated the accumulated ED stay time of all patients. Finally, cardiac arrest occurrence at each specific ED occupancy rate time point was calculated based on the number of cardiac arrest patients at a certain range of ED occupancy rate (e.g., 0.9–1.1) divided by the accumulated time of each ED occupancy rate during the study period.

The primary outcome was to determine the correlation between the four ED occupancy rate measurements and the occurrence rate of unexpected cardiac arrest during ED stay. The secondary outcome was to compare ED mortality according to occupancy rates.

### Statistical analysis

All continuous variables were expressed as median with interquartile range (IQR). The Mann-Whitney *U* test was used to compare the values of continuous variables. Categorical variables were analyzed with the chi-square test or with the Fisher exact test. We assessed the correlation between ED occupancy rate and cardiac arrest occurrence and ED mortality by calculating the Spearman rank correlation coefficient. For all analyses, a two-sided *P* value of < .05 was considered to indicate a statistically significant difference. Statistical analyses were performed by using R version 3.5.0 (R Foundation for Statistical Computing, Vienna, Austria).

## Results

During the study period, 629 adult, non-traumatic patients experiencing OHCA and IHCA were enrolled in our registry. Of these, 187 patients experienced IHCA and 108 patients survived.

The demographic and clinical details of the enrolled patients are summarized in Table 1. The median age was 67.0 years, and patients were predominantly male in both groups. Hypertension (35.3%) was the most common past medical illness in both groups. The survivor group had a more frequent previously diagnosed myocardial infarction (10.2 vs. 1.3%), arrhythmia (20.4 vs. 7.6%), heart failure (16.7 vs. 7.6%), and diabetes (38.0 vs. 19.0%), but less malignancy (25.0 vs. 40.5%) than that of non-survivor. The presumed cardiac source was the leading cause of arrest, and respiratory origin was more common in survivor than in non-survivor (27.8 vs. 15.2%). Additional table represented the total IHCA distribution according to the time and showed no definite patterns (Additional Table 1).

**Table 1:** Demographic data of cardiac arrest patients

	Total IHCA (*n* = 187)	Survivor (*n* = 108)	Non-survivor (*n* = 79)	*P*
Sex (male)	121 (64.7)	69 (63.9)	52 (65.8)	0.08
Age, years	67.0 (55.0–76.0)	67.0 (55.0–74.0)	71.0 (58.5–77.5)	0.18
Medical history				
Cardiac arrest	5 (2.7)	3 (2.8)	2 (2.5)	0.92
Myocardial infarction	12 (6.4)	11 (10.2)	1 (1.3)	0.01
PCI history	16 (8.6)	12 (1.1)	4 (5.1)	0.14
CABG history	7 (3.7)	5 (4.6)	2 (2.5)	0.46
Arrhythmia	28 (15.0)	22 (20.4)	6 (7.6)	0.02
Heart failure	24 (12.8)	18 (16.7)	6 (7.6)	0.07
Hypertension	66 (35.3)	47 (43.5)	19 (24.1)	< 0.01
Diabetes	56 (29.9)	41 (38.0)	15 (19.0)	< 0.01
Chronic lung disease	16 (8.6)	9 (8.3)	7 (8.9)	0.89
Stroke	16 (8.6)	9 (8.3)	7 (8.9)	0.90
Chronic renal failure	22 (11.8)	15 (13.9)	7 (8.9)	0.29
Liver cirrhosis	9 (4.8)	6 (5.6)	3 (3.8)	0.58
Malignancy	59 (31.6)	27 (25.0)	32 (40.5)	0.02
Presumed arrest cause				
Cardiac	61 (32.6)	37 (34.3)	24 (30.4)	0.58
Respiratory	42 (22.5)	30 (27.8)	12 (15.2)	0.04
Bleeding	20 (10.7)	10 (9.3)	10 (12.7)	0.46
Others	64 (33.3)	30 (28.7)	33 (41.7)	0.06

Data are presented as median with interquartile range or percentage

*Abbreviations*: *CABG* coronary artery bypass graft, *CPR* cardiopulmonary resuscitation, *IHCA* in-hospital cardiac arrest, *PCI* percutaneous coronary intervention

Table 2 shows the variables associated with cardiac arrest. Among 187 total IHCA patients, 125 patients (66.8) were successfully ROSC after CPCR. Non-survivor had a higher proportion of DNR after ROSC (36.7 vs. 7.5%). All IHCA events were witnessed, and the median no-flow times were 0 min (IQR 0.0–0.0 min). Pulseless electrical activity was predominant in both groups, and initial ventricular fibrillation rhythm was more common in patients with survivor than with non-survivor (13.0 vs. 2.5%). Regarding the treatment-related factors, the survivor group had more hypothermia, percutaneous coronary intervention, and extracorporeal membrane oxygenation than the non-survivor. Time to first defibrillation, epinephrine injection, and endotracheal intubation was similar in both groups, except for the duration of CPR (6.0 vs. 27.0 min; survivor vs. non-survivor, respectively). Overall survival discharge rate was 24.6%, and 20.3% of patients showed favorable neurologic outcomes at discharge. All ED occupancy rates, including presentation, arrest, maximum, and average, were similar in both groups.

**Table 2:** Characteristics of CPR

	Total IHCA (*n* = 187)	Survivor (*n* = 108)	Non-survivor (*n* = 79)	*P*
ROSC	125 (66.8)	108 (100.0)	47 (59.5)	< 0.01
DNR after ROSC	37 (19.8)	8 (7.5)	29 (36.7)	< 0.01
Initial rhythm^a^				
VF	16 (8.6)	14 (13.0)	2 (2.5)	0.01
Pulseless VT	3 (1.6)	2 (1.9)	1 (1.3)	0.75
PEA	90 (48.1)	50 (46.3)	40 (50.6)	0.56
Asystole	47 (25.1)	22 (20.4)	25 (31.6)	0.08
Unknown	30 (16.0)	19 (17.6)	11 (13.9)	0.50
Hypothermia	17 (9.1)	17 (15.7)	0 (0.0)	< 0.01
PCI	12 (6.4)	10 (9.3)	2 (2.5)	0.06
ECMO	25 (13.4)	21 (19.4)	4 (5.1)	< 0.01
Hospitalization days	2.0 (1.0–14.0)	6.0 (2.0–23.0)	1.0 (1.0–2.0)	< 0.01
Good CPC score at discharge^a^	38 (20.3)	38 (35.2)	0 (0.0)	< 0.01
Survived to hospital discharge	46 (24.6)	46 (42.6)	0 (0.0)	< 0.01
No-flow time, min	0.0 (0.0–0.0)	0.0 (0.0–0.0)	0.0 (0.0–0.0)	0.74
Time from cardiac arrest, min				
First defibrillation	2.0 (0.0–5.0)	2.0 (0.0–4.0)	3.0 (0.0–5.0)	0.59
First epinephrine	0.0 (0.0–1.0)	0.0 (0.0–0.1)	0.0 (0.0–0.1)	0.56
Intubation	0.0 (0.0–8.5)	0.0 (0.0–0.0)	0.0 (0.0–15.0)	0.73
Duration of CPR	10.0 (4.0–30.0)	6.0 (3.0–14.5)	27.0 (9.5–42.0)	< 0.01
ED occupancy rate				
At presentation	0.93 (0.75–1.16)	0.95 (0.76–1.16)	0.94 (0.72–1.09)	0.23
At arrest	0.93 (0.74–1.15)	0.95 (0.75–1.15)	0.94 (0.72–1.10)	0.23
Maximum^b^	1.02 (0.77–1.19)	1.06 (0.83–1.23)	0.95 (0.78–1.16)	0.24
Average^a^	0.93 (0.74–1.15)	0.95 (0.77–1.15)	0.88 (0.73–1.09)	0.24

Data are presented as median with interquartile range or percentage

*Abbreviations*: *CPC* cerebral performance category, *DNR* do-not-resuscitate, *ECMO* extracorporeal membrane oxygenation, *ED* emergency department, *IHCA* in-hospital cardiac arrest, *PCI* percutaneous coronary intervention, *PEA* pulseless electrical activity, *ROSC* return of spontaneous circulation, *VF* ventricular fibrillation, *VT* ventricular tachycardia

^a^CPC scores 1 and 2 were considered as good neurologic outcomes

^b^Maximum and average ED occupancy rate was measured during ED stay before arrest

**Fig. 1:**
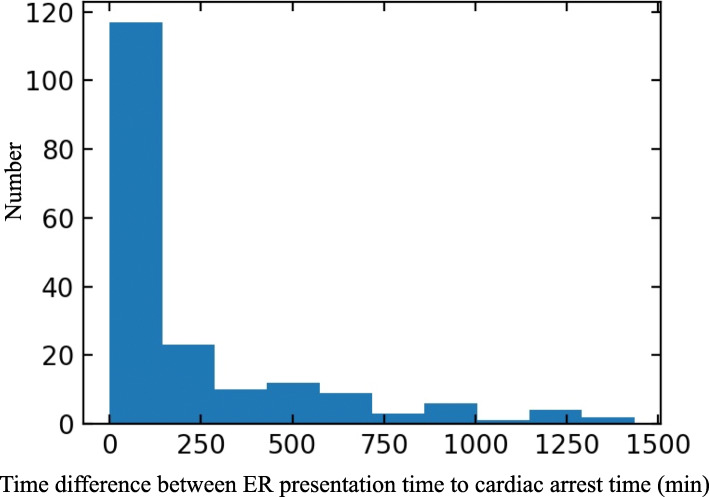
The distribution of ED occupancy and the survival rates

**Fig. 2:**
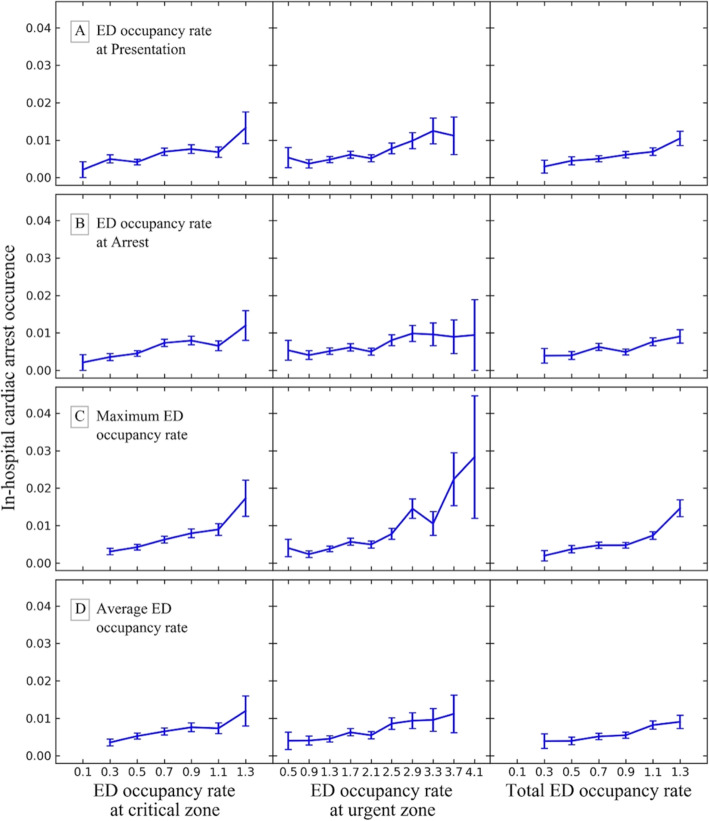
Comparison of the association of ED occupancy rate at critical and urgent zones with cardiac arrest occurrence for four different timings: at presentation, at arrest, maximum, and average on ED stay

**Fig. 3:**
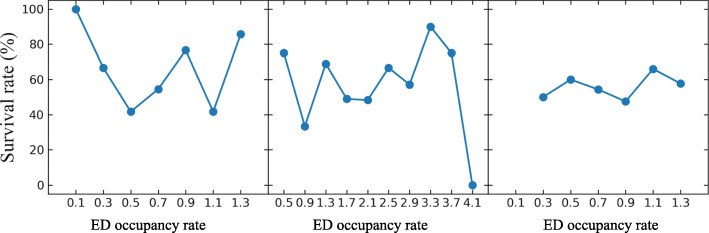
Comparison of the correlation of the maximum ED occupancy rate with the mortality in ED

Figure 1 presents the frequency of cardiac arrest according to time from ED arrival. About two-third cases occurred and the median time was 72.0 min. Figure 2 shows the correlation between the occurrence of cardiac arrest patients and ED occupancy at specific times for each ED zone. Moreover, Fig. 3 represents the association between the death in ED and the maximum occupancy rate. In contrast to the occurrence, mortality in ED was not correlated with the ED occupancy rate.

IHCA occurrence was positively correlated with most of ED occupancy rate except occupancy rate at ED presentation and average during ED stay of the urgent zone (Table 3). All ED occupancy rate in the critical zone was positively correlated with IHCA occurrence, and maximum occupancy rate of IHCA was the most strongly correlated (Spearman’s *ρ* = 1, *P* < .01). Maximum ED occupancy of the urgent zone also yielded a good positive relationship with IHCA occurrence. In the entire ED, all four occupancy rates including presentation, arrest, maximum, and average had strong positive correlations with IHCA occurrence. Table 3 summarizes the results of Spearman rank correlation tests for the relationship between cardiac arrest occurrence and ED occupancy.

**Table 3:** Correlation between ED occupancy rate and cardiac arrest occurrence

	Critical zone ED occupancy rate		Urgent zone ED occupancy rate		Total ED occupancy rate	
	*ρ*	*P*	*ρ*	*P*	*ρ*	*P*
IHCA time point						
At ED presentation	0.86	0.01	0.35	0.33	1.0	< 0.01
At time of cardiac arrest	0.89	< 0.01	0.77	< 0.01	0.96	< 0.01
Maximum ED occupancy rate^a^	1.0	< 0.01	0.94	< 0.01	1.0	< 0.01
Average ED occupancy rate^a^	0.96	< 0.01	0.44	0.20	1.0	< 0.01

*Abbreviations*: *ED* emergency department, *IHCA* in-hospital cardiac arrest

^a^Maximum and average ED occupancy rate was measured during ED stay before arrest

## Discussion

In this study, we compared various types of ED occupancy rate at presentation, at arrest, maximum, and average during ED stay for IHCA occurrence. We found that the maximum ED occupancy rate is most strongly associated with IHCA occurrence. The correlation between ED occupancy rate of the critical zone and IHCA occurrence was stronger than that of the urgent zone. Meanwhile, any ED occupancy rate did not show the relationship with the outcomes of IHCA.

We found that higher maximum ED occupancy rates, which may indicate the most hazardous and chaotic times in the ED, were strongly associated with sudden IHCA occurrence. In a recent observational retrospective study conducted by Chang et al., ED crowding was roughly associated with IHCA occurrence [23]. In this study, ED bed occupancy rate (EDBOR) was used as an overcrowding index by calculating the ratio of the total number of filled beds divided by the total number of licensed ED beds. However, EDBOR is an index that has never been used before and it is difficult to compare with other studies. Moreover, the EDBOR might lead to substantial selection bias because filled beds may not reflect the proportion of critically ill patients at risk of experiencing IHCA in the ED, such as patients waiting for a bed. Ye et al. recently found that there was no association between IHCA events and EDBOR when they calculated the IHCA incidence rate [24].

We also questioned the value of only measuring ED occupancy rate only at ED presentations, which is done in most ED occupancy rate studies, as it might not be sufficient to evaluate the types of overcrowding that ED patients may experience during their ED stay. Other measurements, such as at the time of presentation, arrest, or maximum occupancy, or average ED occupancy rate during ED stay may be better parameters for reflecting overcrowding. To the best of our knowledge, this study is the first study that compared the relationship between IHCA occurrence and ED occupancy rate at these times. Chang et al.’s study used three arbitrary cut-off values of the bed occupancy rate [23], but ED occupancy rate crowding measures should be dealt with as a continuous variable because there is no standard threshold. In our study, we normalized with IHCA incidence per hour for each ED occupancy rate interval (0.2 increment for the critical zone and 0.4 increment for the urgent zone) and found there was a strong association between unexpected IHCA and ED occupancy rate. Maximum ED occupancy might imply the busiest or worst crowding and caused IHCA through multifactorial mechanisms, including decrease surveillance for at-risk patients, decrease medical resources to initial resuscitation, increase turnaround time of laboratory and image workup, or increase medical errors. Meanwhile, our study also proved that not only overall crowding but the portion of critically ill patients dramatically impacts on the occurrence of IHCA. In the Spearman rank correlation test, all rho values of the critical zone were higher than those of the urgent zone. Moreover, the critical zone showed a definite positive correlation with IHCA occurrence at sub-maximal level of ED occupancy rate (≤ 0.9). These trends would imply that even a small number of critical patients could compromise the quality of ED services and surveillance ability of at-risk patients.

We also found that although higher ED occupancy rates showed a positive relationship with the occurrence of unpredicted CA, it was not correlated with ED mortality. Multifactorial reasons could explain this. One of the main reasons was that whenever IHCAs occurred, the available number of medical personnel involved in CPR regardless of the degree of the crowding. This result also could imply that monitoring and trying to control ED occupancy rates could reduce the incidence of IHCA. Based on the ED occupancy rate, the study facility has tried to diminish overcrowding through performing ambulance diversion, shortening the required time for decision-making, and transferring the patients to nearby hospitals whenever there were no available beds in general wards or ICUs to reduce the number of patients waiting for boarding. Similar results were reported in the previous study although they focused on OHCA [14].

## Limitations

Our study had several limitations. First, the retrospective cohort design imposes intrinsic limitations on the study, making it difficult to generalize the findings. Second, we could not exclude various confounding factors to develop cardiac arrest, such as the severity of illnesses before the occurrence of cardiac arrest. In the past, South Korea used the modified Canadian Triage and Acuity Scale for triage; however, the Korean Society of Emergency Medicine reformed this scale and introduced the revised scale called the Korean Triage and Acuity Scale since 2016 [16]. Therefore, our data had no consistent triage scale because the system was changed in the study period. Third, the registry had information only about enrolled patients experiencing IHCA. Therefore, we could not analyze and evaluate the risk factors of IHCA in overcrowded ED. Lastly, this study included a relatively small number of patients at a single center.

## Conclusion

We found that the maximum ED occupancy rate is strongly associated with the IHCA occurrence. Adequate monitoring and active controlling of the maximum occupancy rate would be important to reduce unpredicted cardiac arrest in ED.

## Supplementary information

**Additional file 1: Table S1.** Comparisons of the survival rates between ED occupancy rate at critical and urgent zone according to the time.

## Data Availability

The datasets generated and analyzed during the current study are available from the corresponding author on reasonable request.

## Abbreviations

CPC: Cerebral performance category
CPR: Cardiopulmonary resuscitation
DNR: Do-not-resuscitate
ED: Emergency department
EDBOR: Emergency department bed occupancy rate
IHCA: In-hospital cardiac arrest
IQR: Interquartile range
OHCA: Out-of-hospital cardiac arrest
ROSC: Return of spontaneous circulation
