# Correction to: Maximum emergency department overcrowding is correlated with occurrence of unexpected cardiac arrest

**DOI:** 10.1186/s13054-020-03211-y

**Published:** 2020-08-03

**Authors:** June-sung Kim, Hyun-Jin Bae, Chang Hwan Sohn, Sung-Eun Cho, Jeongeun Hwang, Won Young Kim, Namkug Kim, Dong-Woo Seo

**Affiliations:** 1 Department of Emergency Medicine, University of Ulsan, College of Medicine, Asan Medical Center, Seoul, Republic of Korea; 2 Promedius Inc., Seoul, Republic of Korea; 3 Nursing Department, Asan Medical Center, Seoul, Republic of Korea; 4 Department of Convergence Medicine, University of Ulsan, College of Medicine, Asan Medical Center, 88, Olympic-ro 43-gil, Songpa-gu, Seoul, 05505 Republic of Korea; 5 Department of Emergency Medicine, Biomedical Informatics, University of Ulsan, College of Medicine, Asan Medical Center, 88, Olympic-ro 43-gil, Songpa-gu, Seoul, 05505 Republic of Korea

**Correction to: Crit Care 24, 305 (2020)**

**https://doi.org/10.1186/s13054-020-03019-w**

Following publication of the original article [1], the authors reported an error in Fig. 3 that the indexes of the x-axis in each graph were missing.

The revised Fig. 3 is indicated hereafter.

**Fig. 3:**
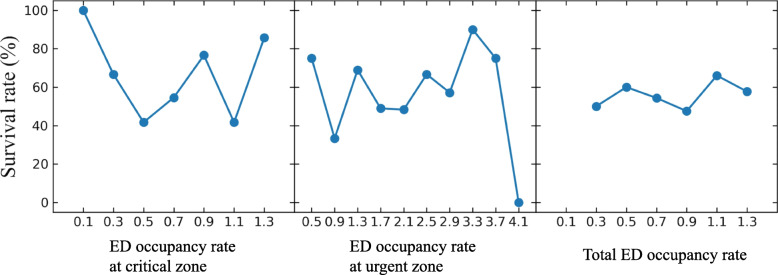
Comparison of the correlation of the maximum ED occupancy rate with the mortality in ED

This has now been included in this correction article.
