# Supplementary material for: Maximum emergency department overcrowding is correlated with occurrence of unexpected cardiac arrest
Source: Crit Care. 2020 Jun 6;24:305. doi: 10.1186/s13054-020-03019-w (PMC7276085; doi:10.1186/s13054-020-03019-w)
Supplement: Supplementary file 1 — Additional file 1: Table S1. Comparisons of the survival rates between ED occupancy rate at critical and urgent zone according to the time. [file 13054_2020_3019_MOESM1_ESM.pdf]

Additional table 1. Comparisons of the survival rates between ED occupancy rate at critical and urgent zone according to the time

Time (o'clock)	IHCA (*n*)	ED occupancy rate at critical zone	ED occupancy rate at urgent zone	Total ED occupancy rate	Survival rate (%)
1-2	6	0.48±0.18	1.33±0.45	0.67±0.22	0.33
3-4	15	0.66±0.25	1.45±0.60	0.73±0.22	0.73
5-6	14	0.68±0.25	1.31±0.54	0.74±0.25	0.5
7-8	14	0.73±0.30	1.81±0.70	0.84±0.28	0.36
9-10	14	0.76±0.27	1.94±0.56	0.92±0.21	0.57
11-12	18	0.83±0.27	2.23±0.77	1.03±0.26	0.67
13-14	16	0.77±0.21	2.45±0.55	1.03±0.20	0.5
15-16	25	0.87±0.23	2.35±0.63	1.07±0.20	0.6
17-18	20	0.82±0.28	2.07±0.58	0.97±0.23	0.5
19-20	16	0.74±0.24	2.29±0.79	1.00±0.24	0.75
21-22	18	0.63±0.20	1.96±0.69	0.88±0.19	0.61
23-24	11	0.78±0.24	2.12±0.78	0.96±0.25	0.64

Abbreviations: ED, emergency department; IHCA, in-hospital cardiac arrest.
